# Clinical epidemiological and laboratory investigation in co-infection with COVID-19 and tuberculosis

**DOI:** 10.1590/S1678-9946202466065

**Published:** 2024-11-11

**Authors:** Ana Carulina Guimarães Belchior, Antônio Martins de Freitas, Grassyelly Silva Gusmao, Evelin Jaqueline Lima dos Santos, Everton Ferreira Lemos, Mauricio Antonio Pompilio, Cláudia Elizabeth Volpe-Chaves, Eliana da Costa Alvarenga de Brito, Everton Falcão de Oliveira, Ana Caroline Blanco Carreiro, Anamaria Mello Miranda Paniago

**Affiliations:** 1 Universidade Federal de Mato Grosso do Sul, Faculdade de Medicina, Programa de Pós-Graduação em Saúde e Desenvolvimento da Região Centro-Oeste, Campo Grande, Mato Grosso do Sul, Brazil; 2 Universidade Estadual de Mato Grosso do Sul, Faculdade de Medicina, Campo Grande, Mato Grosso do Sul, Brazil; 3 Hospital Regional de Mato Grosso do Sul, Campo Grande, Mato Grosso do Sul, Brazil; 4 Unimed, Serviço de Controle de Infecção Hospitalar, Campo Grande, Mato Grosso do Sul, Brazil; 5 Universidade Federal de Mato Grosso do Sul, Hospital Universitário Maria Aparecida Pedrossian, Campo Grande, Mato Grosso do Sul, Brazil; 6 Hospital CASSEMS, Núcleo de Ensino e Pesquisa, Campo Grande, Mato Grosso do Sul, Brazil; 7 Universidade Federal de Mato Grosso do Sul, Faculdade de Medicina, Programa de Pós-Graduação em Doenças Infecciosas e Parasitárias, Campo Grande, Mato Grosso do Sul, Brazil

**Keywords:** Pulmonary tuberculosis, COVID-19, Co-infection, Mycobacterium tuberculosis, SARS-CoV-2

## Abstract

Currently, COVID-19 and tuberculosis (TB) are the deadliest infectious diseases worldwide. Their synergy, form of presentation, morbidity, and mortality are data that have been scarcely explored. Thus, this study aimed to characterize the clinical, epidemiological, and laboratory factors of this co-infection and to analyze the factors associated with the active TB among COVID-19 cases. A case-control study was conducted with a retrospective survey of 21 laboratory-confirmed COVID-19/TB co-infected patients (case group) and 21 COVID-19 patients (control group). The study included participants from eight hospitals in Campo Grande city, capital of Mato Grosso do Sul State, Brazil, from March 2020 to March 2022. Association analysis and binomial logistic regression were employed with statistical significance set at p≤0.05. From the 21 identified cases of COVID-19/TB co-infection, we found a more frequent association with HIV infection than the control-group, without worsening the outcome. COVID-19/TB patients had less dyspnea and less need for mechanical ventilation compared to the cases with COVID-19 only. On the other hand, COVID-19/TB patients had higher levels of C-reactive protein and lower hemoglobin levels, the latter variable was independently associated with COVID-19/TB. Among the clinical differences presented among COVID-19/TB co-infected patients, despite the association with HIV and lower clinical repercussions, only lower hemoglobin levels were associated with COVID-19/TB.

## INTRODUCTION

The threat of contagious infectious diseases is constantly evolving and there is a particular interest in the knowledge about the consequences of the superimposition of viral epidemics (especially SARS-CoV-2) on long-term diseases such as tuberculosis (TB), which continues to be a major disease for public health worldwide, especially in emerging countries^1^.

The first case of Coronavirus disease 2019 (COVID-19) in the Mato Grosso do Sul State, Brazil, was reported in 2020, and since then, there was possibility for both diseases to co-exist. By February 2022, Brazil had recorded 28,245,551 confirmed cases since the beginning of the pandemic, with a 2.3% fatality rate, whereas the state had 500,692 confirmed cases, and recorded 10,319 deaths during this period^2,3^.

The Mato Grosso do Sul State is one of the regions in Brazil with a high incidence of TB (47 cases per 100,000 inhabitants), as well as a high mortality rate (3.1 deaths per 100,000 inhabitants). Only Campo Grande city recorded 5,201.7 new cases of TB in 2022^4^.

TB continues to be an important global public health issue, being the second leading cause of death worldwide due to a single infectious agent, after COVID-19 disease, and has caused almost twice as many deaths as the human immunodeficiency virus (HIV)^5^.

During the COVID-19 pandemic, discussions were raised about the sustainability of TB services and interactions between these two dangerous respiratory pathogens to anticipate this disease impact^6^. Many studies have demonstrated that the significant reallocation of human and material resources within Health Services to combat the COVID-19 pandemic negatively impacted TB control efforts, leading to decreases in diagnosis, treatment, and notification of TB cases^7-10^. However, there has been limited research on the simultaneous occurrence of TB and COVID-19^11^, as well as the challenges in clinical suspicion and diagnosis of TB when it coincides with COVID-19.

The simultaneous occurrence of both diseases can be influenced by immunological changes caused by SARS-CoV-2, which may activate latent TB foci, whether pulmonary or extrapulmonary. Conversely, TB can exacerbate the severity of COVID-19, increasing the likelihood of hospitalization.

Perhaps, many COVID-19 cases also had concurrent, undiagnosed TB. This condition poses significant risks not only to the patient's health but also increases the likelihood of intra-hospital transmission. This study addresses this knowledge gap by investigating the clinical and laboratory variables that could indicate the presence of either pulmonary or extrapulmonary TB among patients with COVID-19.

## MATERIALS AND METHODS

### Location, period, and design of the study

An unmatched case-control study was conducted with a retrospective survey of adult patients with COVID-19 and TB co-infection admitted to a hospital from March 2020 to March 2022, using data obtained from eight public and private hospitals in Campo Grande city, Mato Grosso do Sul State.

### Eligibility criteria and case definition

The inclusion criteria encompassed patients aged 18 years or above who had COVID-19 and/or any clinical form of tuberculosis notified by the Epidemiological Surveillance Centers in the study hospitals, with laboratory confirmation of the diseases.

Cases of active pulmonary tuberculosis notified only by clinical epidemiological criteria were excluded from the study considering the overlap of clinical symptoms and radiological changes between COVID-19 infection and TB.

To define a case of confirmed TB, documented evidence of one of the following criteria was adopted: 1) positive smear microscopy in sputum, tracheal suction and bronchoalveolar lavage, or other biological material, 2) positive molecular assay (TRM-TB, GeneXpert®), or 3) positive culture in sputum, tracheal suction and bronchoalveolar lavage, or other biological material. The TB case could be diagnosed with more than one method and more than one organ could be involved.

For COVID-19, the criteria were documented evidence of one of the following tests: 1) reverse transcription quantitative real-time polymerase chain reaction (RT-qPCR) test with detection of SARS-CoV-2 in a reactive (positive) nasal swab or 2) reactive (positive) SARS-CoV-2 antigen test in a nasal swab.

From these cases, two groups were designed: a) a case group composed of patients with COVID-19 and TB co-infection, both confirmed under laboratory testing and b) a control group containing only patients with COVID-19 confirmed in laboratory, without a diagnosis of TB. The selection for the control group was randomized by simple draw, in the proportion 1:1, not paired, based on the database of patients notified for COVID-19 and confirmed in laboratory.

### Studied variables

The variables analyzed were retrieved from notification forms and medical records and included:

- Demographic Variables: sex, age, and skin color.
- Clinical Variables: pre-existing comorbidities, signs and symptoms, clinical form of tuberculosis, need for mechanical ventilation and renal replacement therapy, and death.
- Laboratory Variables at Admission: diagnostic tests for COVID-19 and tuberculosis, blood count, C-reactive protein (CRP), urea, creatinine, aspartate aminotransferase (AST), and alanine aminotransferase (ALT).

The data were entered into an Excel spreadsheet.

Outcome: the main objective of this study was to identify associations between the aforementioned variables and the presence of active TB among patients with COVID-19.

### Statistical analyses

The frequency and central measures of the variables were compared between cases and controls. For the analysis of associations between categorical variables, the Chi-square test or the Fisher's exact test were employed, depending on the sample size and expected frequencies. For numerical variables, the Mann-Whitney U test was employed to assess differences between groups. Associations between variables were quantified using odds ratios (OR) along with their corresponding 95% confidence intervals.

The binomial logistic regression model was used to assess the association between outcome (TB/COVID-19 comorbidity) and the clinical, laboratory, and covariates data related to the comorbidities of the study participants. Covariates with a p-value lower than or equal to 0.20 in the univariate analysis were included in the multivariate analysis.

Variable selection, along with the control of potential confounding factors, was performed using a stepwise algorithm (considering both backward and forward directions) and the Akaike information criterion (AIC) to determine the best-fitting model. Multicollinearity was assessed both *a priori*, via the correlation matrix of the independent variables, and *a posteriori*, by the variance inflation factor (VIF). As an adjustment measure, the Hosmer and Lemeshow test was used. Statistical significance was set at p ≤0.05 for all hypotheses.

The statistical analysis was conducted using the R software (version 4.3.2, R Foundation for Statistical Computing, Vienna, Austria), using the packages: tidyverse, descr, and generalhoslem.

### Ethics

All ethical procedures were respected, following resolution Nº 466/2012 of the Brazilian National Council of Health, and the project was approved by the research ethics committees of Universidade Federal de Mato Grosso do Sul (protocol Nº 61665622.2.000.0021) and Associacao Beneficente Santa Casa de Campo Grande (protocol Nº 61665622.2.3001.0134).

## RESULTS

During the period and at the locations of the study, 16,006 cases of COVID-19 were notified. Among these cases, 178 were also notified for TB. Only 21 cases out of the 178 notified cases of co-infection were selected, as they met the inclusion criteria, with laboratory testing, and these were included in the descriptions and analyses of the case group.

The co-infections were diagnosed in five of the eight hospital institutions, with 20 hospital admissions in the public sector and only one in the private sector.

The groups showed no significant differences in their baseline characteristics, except for the fact that HIV infection was observed only in the case group (p<0.001) (Table 1).

**Table 1:** Basal clinical epidemiological characteristics of 21 patients with COVID-19 and active tuberculosis (case group) and 21 patients with COVID-19 without tuberculosis (control group).

Parameter		Case group n (%)	Control group n (%)	OR [95%CI]	p-value
**Demographic characteristics**					
	Age (years) mean ± SD	44.8 ±12.7	44.8±2.8	1.00 [0.95;1.05]	0.134
	Male	14 (66.7)	14 (66.7)	1.00 [0.27;3.75]	1.000
	Non-White	15 (71.4)	10 (50.0)	2.42 [0.67;9.49]	0.160
**Co-morbidities**					
	Systemic arterial hypertension	3 (14.3)	16 (76.2)	0.29 [0.05; 1.24]	0.159
	Diabetes mellitus	4 (19.0)	5 (23.8)	0.76 [0.16; 3.52]	1.000
	HIV	9 (42.9)	–	**-**	**0.001**
**Confirmation of COVID-19**					
	RT-PCR	18 (85.7)	20 (95.2)		0.500
	Antigen test	3 (14.2)	1 (4.8)		

In bold: p =< 0.05, SD = standard deviation; OR [95% CI] = odds ratio [95% confidence interval]

Most patients with TB were classified as new cases, with positive smear microscopy and pulmonary involvement. In total, seven patients had previously been diagnosed with TB but had not completed their treatment, characterizing abandonment. However, the timing of their initial diagnosis was not registered in the medical records (Table 2).

**Table 2:** Characteristics of the active tuberculosis in 21 patients with COVID-19.

Characteristics of the tuberculosis		n (%)
**Type of case**		
New case		13 (61.9)
Recurrence		1 (4.7)
Reentry after abandonment		7 (33.3)
**Diagnostic confirmation** *		
	Smear microscopy	12 (57.1)
	Rapid molecular assay	8 (38)
	Culture	4 (19)
**Form of presentation**		
	Exclusive pulmonary	15 (71)
	Pulmonary and extra-pulmonary	3 (14.5)
	Exclusive extra-pulmonary	3 (14.5)
**Involved extra pulmonary organ** **		
	Pleura	3 (50)
	Lymph node	2 (33.3)
	Central nervous system	1 (16.7)
**Treatment instituted during hospitalization**		
	RHZE	18 (85.7)
	Not treated	3 (14.3)

* the case may have been identified by more than one method

** more than one organ could be involved; AFB = acid-fast bacillus; RHZE = Rifampicin, Isoniazid, Pyrazinamide, and Ethambutol. The case may have been diagnosed with more than one method.

Dyspnea was the only symptom with a significant difference, more frequent in cases with isolated COVID-19. The control group also required mechanical ventilation more frequently than the case group (Table 3).

**Table 3:** Comparison of the frequency of symptoms at admission and among 21 patients with COVID-19 and active tuberculosis (case group) and 21 patients with COVID-19 without tuberculosis (control group).

Parameter		Case group n (%) n=21	Control group n (%) n=21	cOR [95%CI]	p-value
**Symptoms**					
	Fever	8 (38.1)	7 (33.3)	1.22 [0.34;4.53]	1.000
	Hypoxia	8 (38.1)	2 (9.5)	5.36 [1.09;44.0]	0.070
	Cough	7 (33.3)	10 (47.6)	0.56 [0.15;1.97]	0.346
	Dyspnea	6 (28.6)	13 (61.9)	0.26 [0.06;0.92]	**0.030**
	Chest pain	2 (9.5)	2 (9.5)	1.00 [0.10;10.4	1.000
	Headache	2 (9.5)	2 (9.5)	1.00 [0.10;10.4]	1.000
	Dizziness	2 (9.5)	–	–	0.490
	Diarrhea	1 (4.8)	–	–	1.000
	Nausea / Vomiting	1 (4.8)	1 (4.8)	1.00 [0.02;40.9]	1.000
	Syncope	1 (4.8)	1 (4.8)	1.00 [0.02;40.9]	1.000
	Bradycardia	1 (4.8)	–	–	1.000
	Odynophagia	1 (4.8)	3 (14.3)	0.33 [0.01;3.15]	0.610
**Factors related to the severity**					
	Days of hospitalization median [IQR]	21 [19]	13 [20]		0.134
	Days of hospitalization < 20 days*	9 (42.9)	14 (66.7)	0.64 [0.22;1.91]	0.121
	Mechanical ventilation	1 (4.8)	12 (57.1)	0.05 [0.00;0.29]	**<0.001**
	Hemodialysis	2 (9.5)	2 (9.5)	1.00 [0.13;7.85]	1.000
	Death	5 (23.8)	5 (23.8)	1.00 [0.23;4.42]	1.000

cOR [95%CI] = crude Odds Ratio [95% confidence interval]. In bold: p =< 0.05, IQR = interquartile range;

* patients discharged before 20 days of hospitalization.

In the case group, patients with HIV were younger, and among them, no variable associated with disease severity was observed (Table 4). Upon admission, patients in the case group showed lower serum hemoglobin and higher C-reactive protein (CRP) and creatinine values (Table 5).

**Table 4:** Epidemiological and clinical analyses of 21 patients with COVID-19 and active tuberculosis, according to the presence of HIV infection.

Parameter		without HIV n (%) n=12	with HIV n (%) n=9	p-value
**Demographic**				
	Age (in years) mean ± SD	52.6±8.4	34.3±9.6	**<0.001**
	Male sex	9 (75.0)	5 (55.6)	0.397
	Non-White	9 (75.0)	6 (66.7)	1.000
**Clinical**				
	Hypertension	2 (16.7)	1 (11.1)	1.000
	Diabetes mellitus	4 (33.3)	–	0.100
	Days of hospital admission mean ± SD	20.58 ±16.6	27.9±30.0	0.300
	Days of hospital admission < 20 days	6 (50.0)	3 (33.3)	0.660
	Mechanical ventilation	1 (8.3)	–	1.000
	Hemodialysis	1 (9.1)	1 (11.1)	1.000
	Death	3 (25.0)	2 (22.2)	1.000

In bold: p =< 0.05, SD = standard deviation

**Table 5:** Comparison of the laboratory exams of 21 patients with COVID-19 and active tuberculosis (case group) and 21 patients with COVID-19 without tuberculosis (control group).

Exams	Case group Median (IQR)	Control group Median (IQR)	p-value
Hemoglobin (g/dl)	9.90 (4.57)	13.9 (2.00)	**<0.001**
Leukocytes (mm^3^)	6,805 (8.268)	8,760 (4,330)	0.155
Platelets (mm^3^)	245,000 (265,250)	191,000 (99,000)	0.725
CRP (mg/dL)	47.8 (138.00)	16.6 (17.2)	**<0.001**
Potassium (mmol/ L)	4.00 (0.47)	4.10 (0.43)	0.426
AST (U/L)	49.4±50.7	50.9±37.7	0.354
ALT (U/L)	29.3±18.4	43.6±43.4	0.253
Creatinine (mg/dL)	1.18±1.49	1.20±0.80	0.012
Urea (mg/dL)	45.2±49.4	42.5±25.9	0.206

In bold: p =< 0.05, SD = standard deviation, IQR = interquartile range, CRP = C-reactive protein, aspartate aminotransferase (AST), alanine aminotransferase (ALT)

To understand how covariates impact the presence of TB in COVID-19 patients, a logistic regression model was used. The final model includes covariates HIV, hyporexia, hemoglobin, CRP, and hypertension. However, only hemoglobin was significantly associated with COVID-19/TB. A negative β coefficient suggests that lower hemoglobin levels lead to higher risks of TB/COVID-19 comorbidity. The adjusted odds ratio of 0.49 suggests that for each unit decrease in hemoglobin, the chance of comorbidity rises by 51% (1 − 0.49 = 0.51) (Table 6). The other variables contributed to the adjustment of the model but were not associated with the outcome. The non-significant Hosmer and Lemeshow test (p = 0.327) suggested that the model was well adjusted to VIF for the explanatory variables, indicating the absence of multicollinearity.

**Table 6:** Data from the multivariable logistic regression analysis (outcome = TB/COVID-19 comorbidity).

Covariables	β Coefficient (Standard Error)	aOR (95% CI)
Intercept	10.74* (4.79)	-
HIV (Yes)	18.03 (2624)	-
Hemoglobin	−0.72* (0.35)	0.49 (0.20, 0.87)
Systemic arterial hypertension (Yes)	−3.36 (0.00)	0.03 (0.00, 0.68)
C reactive protein	−0.02 (0.01)	1.02 (1.00, 1.04)
Hypoxia	−2.64 (1.60)	0.07 (0.00, 1.19)

aOR = adjusted odds ratio; CI = confidence interval;

* p-value < 0.05

## DISCUSSION

The study identified a relatively small sample of co-infected patients, 21 in total, representing 11% of the patients notified for TB by the Epidemiological Surveillance Centers in the study hospitals.

The selection of cases with laboratory confirmation was only due to both diseases sharing similar clinical symptoms—such as cough and fever—and not presenting totally specific tomographic changes, i.e., the use of clinical epidemiological and/or clinical radiological criteria could lead to biases in the comparative study.

Considering these observations, we highlight that the total number of co-infected people may also be underestimated due to the pandemic, when the diagnosis of pulmonary TB was often delayed due to atypical imaging findings^8^. On the other hand, it is important to consider that some patients were diagnosed with TB based solely on smear microscopy, which might have included cases of infection with non-tuberculous mycobacteria. However, the prevalence of non-tuberculous mycobacteria in both pulmonary and extra-pulmonary samples from suspected TB cases is uncommon^12^.

In Brazil, the COVID-19 pandemic reduced the TB detection rates in all Brazilian regions and in 81.5% of Brazil's federative units states. Approximately 60% of the municipalities showed stabilization or decrease in the detection rates^9,10^.

Another strategy employed to reduce selection bias and/or confounding factors was to encompass the main hospitals in the Campo Grande city, spread across different regions of the capital. Among these facilities, some provide exclusively private care, whereas others provide healthcare via the Unified Health System (SUS), causing heterogeneity of the sample data.

Regarding clinical epidemiological baseline characteristics, the groups presented similar data except for two aspects: symptoms of dyspnea reported more frequently in the control group and presence of people living with HIV, present only in the case group. The other clinical symptoms reported prior to the hospital admission were similar between groups.

Dyspnea was more frequent in the control group, nonetheless its absence was not independently associated with TB/COVID-19, suggesting that the observed association may have been influenced by a confounding factor. Since all patients were hospitalized, this likely reflects the primary reason for hospitalization in patients with COVID-19, respiratory failure, while those with TB associated may have been admitted for other reasons, such as general health deterioration^13^. This also justifies the fact that the control group showed more patients who required mechanical ventilation.

However, considering that TB cases also were infected with COVID-19, there could be some interaction between these infections that contributed to the lower frequency of this symptom.

The association between TB and HIV is not unknown. In 2019, 8% of the estimated 10 million people who developed TB worldwide were HIV positive^14^. In Brazil, in 2020, the incidence of HIV infection among new notified TB cases was 10%, and Mato Grosso do Sul State was one of the 10 states that surpassed this national proportion, accounting for 11% of the co-infection by TB-HIV^4^. Quite possibly, the high frequency of HIV observed in the study may be related to one of the hospitals being a reference hospital for HIV/AIDS treatment. Given that almost half TB patients were also HIV-positive, this may have influenced the comparison of clinical and laboratory variables between cases and controls, as some differences could be due to HIV infection rather than TB, introducing potential confounding factors. However, in the multivariate analysis, HIV infection was not statistically significant, suggesting that, in this dataset, HIV did not have a meaningful association with the outcome after adjusting for other factors. Nonetheless, the inclusion of HIV in the model was important to control for its potential confounding effect, ensuring proper model adjustment and minimizing bias. However, the findings reinforce the need to investigate TB in patients with COVID-19 and HIV systematically.

HIV infection did not result in longer hospital stay, increased use of mechanical ventilation, need for hemodialysis, or increased mortality when compared to the patients with or without the triple infection (COVID-19/TB/HIV). These findings could be explained by the fact that the patients with triple infection were younger, but other studies have also identified that they do not change the clinical outcome of the patients in the presence of COVID-19 and HIV infection^15,16^.

Among those diagnosed with TB, one third had a prior TB diagnosis but abandoned treatment. TB was mainly diagnosed using the smear microscopy method and treated with RHZE combination (Rifampicin, Isoniazid, Pyrazinamide and Ethambutol). The pulmonary presentation was the most common, and the pleura was the most involved extra-pulmonary organ. These findings corroborate previous data on patients with TB alone^5^.

The large number of cases diagnosed during hospitalization raises the question of whether COVID-19 infection predisposed the emergence of TB, especially considering that approximately 10% of the people with latent pulmonary TB infection progress to active disease throughout their lives, particularly in the first five years of infection^2^, and that the main risk factor for progression to active disease is immunological impairment.

Thus, HIV-infected individuals, patients with chronic renal failure or undergoing immunosuppressive treatment are at greater risk of progression to active disease^17,18^. Other viral infections have also been reported as predisposing to the activation of this disease, such as infection by human T-lymphotropic virus (HTLV)^19^ and viral hepatitis^20^.

Influenza episodes increase the risk of developing active TB in individuals with latent TB infection^21^. Moreover, co-infection with influenza was associated with an increased bacterial load of Mycobacterium tuberculosis and a negative modulation of immune responses specific to tuberculosis^22^. Similarly, the interaction between SARS-CoV-1 and TB has been documented, highlighting the potential viral infections hold to exacerbate TB risk^23^.

The immunosuppressive effects observed in several viral infections, including SARS-CoV-1, suggest a similar risk with SARS-CoV-2, raising the possibility that COVID-19 could lead to the reactivation of latent TB in vulnerable individuals.

After SARS-CoV-2 enters the host, it targets the respiratory epithelial and alveolar cells, interacting with innate immune cells, such as macrophages that may be infected with M. tuberculosis, secreting cytokines and activating other cells of the immune system. An exuberant response can generate lung lesion and weaken the innate immune response, leading to increased adhesion, growth, and dissemination of mycobacteria^24^.

Although it is imperative to control the COVID-19 pandemic, the medical community must not forget the other endemic diseases that impact populations. TB is one of these diseases, and healthcare providers must pay attention to any clinical subtleties to assure timely and accurate diagnosis.

Other studies also highlight the importance of providing opportunities for diagnosis of lung diseases that can present many clinical, laboratory, and imaging findings similar to COVID-19^25-27^.

Regarding complications, the presence of co-infection did not increase hospital length of stay, evolution to hemodialysis, or mortality when compared to the control group. However, the evolution to mechanical ventilation was lower in the co-infected patients, once again demonstrating the importance of studies that analyze the association of radiological, clinical, and anatomy-pathological findings to comprehend this role.

Concerning laboratory findings, the patients in the case group had higher reactive CRP values, similar to that observed in another study^28^, which can be justified by the greater ongoing inflammatory process due to the presence of two associated infections or even three, considering that TB cases showed more HIV infections. However, in the multivariate analysis, CRP was not independently associated with TB/COVID-19 comorbidity, suggesting that other factors may act as confounding variables.

These patients also had lower serum hemoglobin levels than the control group, very possibly due to the anemia of the chronic disease, which is the main disease found in patients with TB^29-31^, and to the fact that some patients may experience reversal of the condition after 60 days of anti-tuberculosis therapy^32^. These findings are consistent with those of another Brazilian study, which also showed that patients with co-infection have lower hemoglobin levels^33^.

Hemoglobin was the only variable that was independently associated with co-morbidity. Thus, in patients with COVID-19, a decrease in hemoglobin increases the chance of co-existing TB, suggesting that TB should be considered in these cases. Although anemia can also occur during COVID-19^34^ infection, it tends to be more pronounced in TB due to the chronic nature of the disease. This is because the chronic inflammatory response in TB interferes with iron homeostasis, reducing the availability of iron for erythropoiesis^35^.

This study shows some limitations. The data were collected retrospectively from medical records, which may have led to incomplete or inadequately registered symptoms. Additionally, we were unable to select a larger control group (such as 1:2 or 1:3) because the medical records of patients without TB often lacked sufficient data. Another limitation is that the multivariate analysis was performed with a small sample and should be cautiously interpreted. Thus, it is possible that other variables associated with this comorbidity were not identified.

## CONCLUSION

In conclusion, the clinical and laboratory similarities between the case and the control groups highlight the challenge of identifying TB cases among COVID-19 patients. Therefore, given the overlap in respiratory symptoms, TB should be systematically investigated in all COVID-19 patients, especially those with reduced hemoglobin.

This study reinforces the need for continuous surveillance on TB, especially in countries with high infectious burden, such as Brazil. Further studies are necessary to explain the characteristics and outcomes.
